# Integrative Functional, Molecular, and Transcriptomic Analyses of Altered Intrinsic Timescale Gradient in Depression

**DOI:** 10.3389/fnins.2022.826609

**Published:** 2022-02-17

**Authors:** Shaoqiang Han, Ruiping Zheng, Shuying Li, Bingqian Zhou, Yu Jiang, Caihong Wang, Yarui Wei, Jianyue Pang, Hengfen Li, Yong Zhang, Yuan Chen, Jingliang Cheng

**Affiliations:** ^1^Department of Magnetic Resonance Imaging, The First Affiliated Hospital of Zhengzhou University, Zhengzhou, China; ^2^Key Laboratory for Functional Magnetic Resonance Imaging and Molecular Imaging of Henan Province, Zhengzhou, China; ^3^Engineering Technology Research Center for Detection and Application of Brain Function of Henan Province, Zhengzhou, China; ^4^Engineering Research Center of Medical Imaging Intelligent Diagnosis and Treatment of Henan Province, Zhengzhou, China; ^5^Key Laboratory of Magnetic Resonance and Brain Function of Henan Province, Zhengzhou, China; ^6^Key Laboratory of Brain Function and Cognitive Magnetic Resonance Imaging of Zhengzhou, Zhengzhou, China; ^7^Key Laboratory of Imaging Intelligence Research Medicine of Henan Province, Zhengzhou, China; ^8^Department of Psychiatry, The First Affiliated Hospital of Zhengzhou University, Zhengzhou, China

**Keywords:** first-episode depression, gene expression profiling, fMRI, intrinsic timescale gradient, neurotransmitter

## Abstract

The pathophysiology and pharmacology of depression are hypothesized to be related to the imbalance of excitation–inhibition that gives rise to hierarchical dynamics (or intrinsic timescale gradient), further supporting a hierarchy of cortical functions. On this assumption, intrinsic timescale gradient is theoretically altered in depression. However, it remains unknown. We investigated altered intrinsic timescale gradient recently developed to measure hierarchical brain dynamics gradient and its underlying molecular architecture and brain-wide gene expression in depression. We first presented replicable intrinsic timescale gradient in two independent Chinese Han datasets and then investigated altered intrinsic timescale gradient and its possible underlying molecular and transcriptional bases in patients with depression. As a result, patients with depression showed stage-specifically shorter timescales compared with healthy controls according to illness duration. The shorter timescales were spatially correlated with monoamine receptor/transporter densities, suggesting the underlying molecular basis of timescale aberrance and providing clues to treatment. In addition, we identified that timescale aberrance-related genes ontologically enriched for synapse-related and neurotransmitter (receptor) terms, elaborating the underlying transcriptional basis of timescale aberrance. These findings revealed atypical timescale gradient in depression and built a link between neuroimaging, transcriptome, and neurotransmitter information, facilitating an integrative understanding of depression.

## Introduction

As one of the leading disabling diseases worldwide (Murray et al., 2012), depression affects approximately 350 million people each year (Schmaal et al., 2017). Recent studies point out that imbalance of the excitation–inhibition (E/I) underlays the pathophysiology and pharmacology of the depression (Voineskos et al., 2019). Imbalance of E/I ratio hypothetically results in the aberrance of hierarchically organized intrinsic neural timescales (Kiebel et al., 2008) that support synchronizing large-scale brain networks usually measured with resting-state functional connectivity (rsFC; Buzsáki and Draguhn, 2004). Accordingly, the intrinsic timescale gradient is theoretically altered in depression that remains unknown yet.

The brain regions are hierarchically organized into increasing polyfunctional neural circuits embodied in topographic gradients of molecular, cellular, and anatomical properties (Huntenburg et al., 2018). Emerging through hierarchically organized feature (Burt et al., 2018) such as pyramidal cell dendritic spine density (Elston, 2003), long-range interactions (Wang, 2020), and gene expression gradients (Fulcher et al., 2019), intrinsic neural dynamics (or intrinsic timescale gradient) are also hierarchically organized, supporting a hierarchy of cortical functions (Kiebel et al., 2008; Hasson et al., 2015). Brain dynamics is also hierarchically organized along spatial gradients extending from sensorimotor regions to association cortex (Hasson et al., 2008) supporting functional communications between brain regions (Cocchi et al., 2016). Regions with longer “temporal receptive windows” are subsequently found to exhibit more slowly changing activity and *vice versa* (Hasson et al., 2015). In particular, regions such as prefrontal areas and parietal areas, densely interconnected central regions, have longer timescales compared to peripheral sensory areas (Chaudhuri et al., 2015) for the reason that prolonged neural timescale is needed to enable these high-order brain regions to integrate various information for robust sensory perception (Hasson et al., 2008), stable memory processing (Bernacchia et al., 2011), and decision-making (Cavanagh et al., 2016). By developing a large-scale biophysical model, Chaudhuri et al. (2015) elaborate that intrinsic timescale gradient depends crucially on recurrent network activity. Aberrance of neural timescales is supposed to the result of imbalance of the excitation–inhibition (E/I) ratio (Wengler et al., 2020). Wengler et al. (2020) find evidence for distinct hierarchical aberrance in timescale gradient as a function of hallucination and delusion, supporting glutamatergic and dopamine theories of psychosis (Corlett et al., 2011; Jardri et al., 2016). Imbalance of the E/I is also implicated in the pathophysiology and pharmacology of the depression (Covington et al., 2010; Voineskos et al., 2019) and the mechanism of fast-acting antidepressant is related to E/I rebalance (Li, 2020). Although the intrinsic timescale gradient should be altered in depression theoretically, it remains unknown yet.

Brain function such as rsFC is also modulated by genetic factors (Richiardi et al., 2015; Fornito et al., 2019; Richiardi et al., 2015) and coupled to neurotransmitters (Stagg et al., 2014; Kringelbach and Cruzat, 2020). Twin studies show that functional connectivity within the default-mode network and topological measures in the human brain are moderate to highly heritable (Glahn et al., 2010; Fornito et al., 2011; van den Heuvel et al., 2013). Recently, Allen Human Brain Atlas (AHBA) (Hawrylycz et al., 2012), a newly proposed brain-wide gene expression atlas, provides the possibility of bridging the gap between transcriptome and large-scale connectome organization (Fornito et al., 2019). Following the work of Richiardi et al. (2015) where they find that the transcriptome profile similarity within networks is higher than that between networks, a number of studies begin to explore the transcriptional basis of macroscopic neuroimaging phenotypes (Krienen et al., 2016; Vértes et al., 2016; Anderson et al., 2018; Li and Seidlitz, 2021). Recently, Zhu et al. (2021) find that spatial distribution of functional connectivity strength is modulated by genes enriched for terms such as synaptic transmission in health (Zhang et al., 2021). In depression, Li et al. (2021) identify that altered morphometric similarity network is correlated with transcriptional signatures (Li and Seidlitz, 2021). In addition, rsFC is found to be coupled to neurotransmitter transporters/receptors (Stagg et al., 2014; Kringelbach and Cruzat, 2020). Dysconnectivity in schizophrenia is linked to altered neurotransmission (Landek-Salgado et al., 2016; Limongi et al., 2020). Chen et al. (2021) find that abnormal functional topography of brain networks is associated with the dopaminergic and serotonergic systems underlying cognitive decline in schizophrenia by investigating the molecular architecture facilitating a link to treatment. The variation of timescales is hypothesized to arise from local biophysical properties of neurons across the cortical hierarchy, such as the density of glutamate receptors, calcium channels, and regulators of synaptic depression and facilitation (Zucker and Regehr, 2002; Wong and Wang, 2006). Investigating the molecular and transcriptional basis of altered intrinsic timescale gradient in depression helps to advance our understanding of how alterations at microscale architecture drive macroscale neuroimaging aberrance in depression.

In this study, we aimed to explore altered intrinsic timescale gradient and its underlying molecular and transcriptional signatures bridging the gap between molecular mechanism and macroscopic neuroimaging phenotypes in depression. First, we presented replicable landscape of intrinsic timescale gradient and its association with commonly used functional indicators including amplitude of low-frequency fluctuation (ALFF) and functional connectivity density (FCD) in two independent Chinese Han cohorts. Second, we investigated altered intrinsic timescale gradient in different stages of depression according to illness duration. Third, we inquired molecular and transcriptional basis of altered intrinsic timescale gradient in depression. Fourth, a functional enrichment analysis was performed to inquire ontological pathways of timescale aberrance-related genes in depression.

## Materials and Methods

### Datasets

Two independent Chinese Han datasets were used in this study. The first dataset come from the Southwest University Adult Lifespan Dataset (SALD) study including 494 healthy participants (female: male, 308:187, 19--80 years old). The second dataset included 121 HCs and 191 patients with depression. The resting-state functional MRI data were acquired and preprocessed using Data Processing & Analysis for Brain Imaging (DPABI)^1^ (Yan et al., 2016). The details about dataset description, scan acquisition, and preprocessing procedures were included in Supplementary Methods. The study was approved by the research ethical committee of the First Affiliated Hospital of Zhengzhou University.

### Calculation of Timescales and Its Association With Functional Connectivity Density and Amplitude of Low-Frequency Fluctuation

Based on previous studies (Watanabe et al., 2019; Raut and Snyder, 2020), we calculated intrinsic neural timescales by calculating the magnitude of autocorrelation of the resting-state brain signals. There were two different definitions of timescales. First, the timescale was defined as the sum of positive autocorrelation function (ACF) values and then multiplied by the repetition time (TR) (Watanabe et al., 2019). The results reported in the next steps were based on this definition. The second was defined as the half of the full width at half maximum of the ACF (Raut and Snyder, 2020). To inquire the relationship between these two definitions, spatial correlation was obtained between the two mean timescale maps across healthy subjects in dataset 1 and healthy subjects in dataset 2, respectively. In addition, we also compared spatial correlation between the altered timescale maps of depression using these two definitions (see below).

As a newly proposed index, factors affecting landscape of timescales remained unclear. To explore these factors, we investigated whether factors such as gender (female vs. male), age, motion movement, and education level could affect intrinsic timescale gradient (Supplementary Methods). The timescales were calculated using custom MATLAB code publicly available at https://github.com/RaichleLab.

To intuitively elucidate the intrinsic timescale gradient measured, we inquired the association between intrinsic timescale gradient with other common functional indexes such as ALFF and FCD. For FCD maps, local, long-range, and global FCD maps were calculated (Tomasi and Volkow, 2010) where correlation threshold was determined by significance of single functional connection (*p*-value). A functional connection (correlation coefficient) was considered significant if its *p* < 0.05 (Bonferroni corrected). The obtained FCD maps were transformed to *z*-scores by subtracting the mean value and dividing by the standard deviation across gray matter voxels. The ALFF maps were calculated using resting-state functional magnetic resonance imaging data processing toolbox (REST) (Song et al., 2011), and normalized ALFF maps (dividing the mean value across gray matter voxels) were chosen for the following steps.

Dominance analysis was used to quantify the association between mean FCD (including local, long-range and global FCD), and mean ALFF maps to landscape of timescales across healthy subjects in each dataset (Budescu and David, 1993; Azen and Budescu, 2003).^2^ The details are provided in Supplementary Methods.

All of the above analysis steps were done in the discovery cohort (dataset 1) and validated in the replication cohort (HCs of dataset 2); results reported were based on the discovery cohort, unless stated otherwise.

### Altered Intrinsic Timescale Gradient in Depression

Then, we explored whether intrinsic timescale gradient was altered in depression. The altered timescale was obtained by using two-tailed two-sample *t*-test equipped in SPM 12 where gender, age, mean FD, SNR0, and educational level were included as covariates. To explore whether aberrance of intrinsic timescale gradient was stage-dependent for the reason that mental disorders were found to present progressive brain structural alterations (Koutsouleris et al., 2014; Treadway et al., 2015; Cao et al., 2017; Zhang et al., 2017; Yüksel et al., 2018), patients with depression were further divided into three stages according to illness duration (Stage 1: 0 ≤ illness duration ≤12 months; Stage 2: 12 <illness duration ≤24 months; Stage 3: illness duration ≥24 months). Moreover, we also compared timescales in patients whose illness duration is less than 3/6 months to inquire whether timescale aberrance emerged from the beginning of the disease. Results reported in this study were corrected for multiple comparison (voxel-wise *p* < 0.001, cluster-level *p* < 0.05; GRF correction). To further explore the relationship between two definitions of timescales, the same statistical procedures were done in the second definition of timescales (half maximum of the ACF).

### Spatial Correlation Between Altered Timescales of Depression With Receptor/Transporter Densities

To explore association between depression-induced changes in timescales and expression of a specific receptor/transporter, we evaluated the spatial relationship between altered timescales and the distribution of receptors/transporters. The timescale difference map was spatially correlated with PET/SPECT maps in JuSpace toolbox^3^ (Dukart et al., 2021). The default neuromorphometrics atlas excluding white matter and cerebrospinal fluid regions was used. Dopamine (D1 and D2), serotonin receptors (5-HT1a, 5-HT1b, and 5-HT2a), transporters (dopamine transporter and serotonin reuptake transporter 5-HTT), F-DOPA (a reflection of presynaptic dopamine synthesis capacity), the GABAergic receptor, and the noradrenaline transporter (NAT) were investigated. The correlation results were adjusted for spatial autocorrelation of local gray matter probabilities, and the significance of results was computed using permutation statistics (Dukart et al., 2021). To exclude the effect of atlas choice on our results, we used another two atlas (268 and 246 regions) (Shen et al., 2013; Fan et al., 2016) to validate these results.

What is more, to inquire whether the association with receptor/transporter densities was specific to altered timescales, we calculated spatial correlation between ALFF differences of depression with PET/SPECT maps using default neuromorphometrics atlas. The ALFF was chosen for the reason that it was widely used in resting-state fMRI studies.

### Cortical Gene Expression Related to Altered Timescale of Depression

Inspired by a previous study (Reardon and Seidlitz, 2018), we ranked genes based on the spatial correlation between gene expression pattern and the voxel-wise unthresholded *t*-statistic map of timescale difference in depression. The gene expression data come from theAHBA^4^ (Hawrylycz et al., 2012), obtained from six adult human brains (Hawrylycz et al., 2012). Details and preprocessing procedures of AHBA were included in Supplementary Methods. The preprocessed AHBA used in this study comes from the Brain Annotation Toolbox (BAT)^5^ (Liu et al., 2019). Because only two right hemisphere data were included in the AHBA, we only considered the left hemisphere in our analysis (Arnatkeviciute et al., 2019). The correlation results were considered significant if |*r*| > 0.2 and *p* < 0.05 (FWE corrected). Finally, the positive and negative correlation gene lists were identified with timescale aberrance.

### Enrichment Pathways Associated With Altered Timescales of Depression

We performed the gene ontology (GO) and Kyoto Encyclopedia of Genes and Genomes (KEGG) pathways with the genes presenting significant spatial correlation with altered timescales of depression using Metascape (Zhou et al., 2019). Results reported here were corrected by the FDR (*p* < 0.05). This procedure was done in positive and negative correlation genes separately.

As done in a previous study (Li and Seidlitz, 2021), we further investigated shared enrichment terms between previously reported polygenic risk for depression and the timescale-related gene list (Wray et al., 2018; Howard and Adams, 2019). A multi-gene list meta-analysis was carried out between the timescale aberrance-related gene list and the gene list provided by these two studies.

## Results

### Clinical Demographics

The clinical demographics of subjects in dataset 1 and dataset 2 are included in Supplementary Tables 1, 2.

### The Landscape of Resting-State Timescales and Its Association With Functional Connectivity Density and Amplitude of Low-Frequency Fluctuation

**FIGURE 1:**
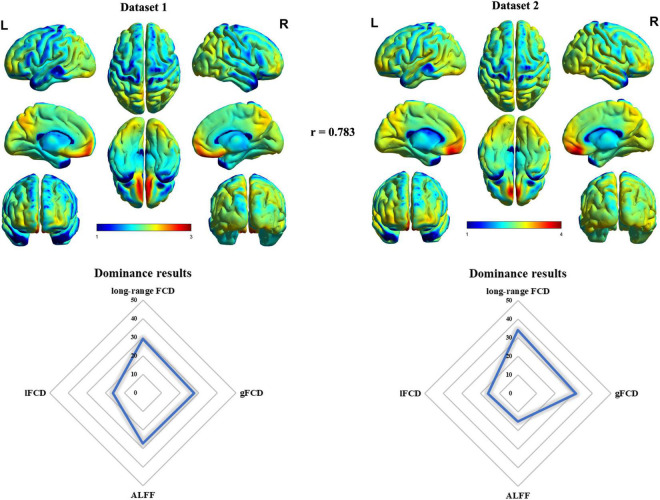
The landscape of timescales and its association with ALFF and FCD. The “r” meant the spatial correlation between mean timescales maps in dataset 1 and dataset 2. The number in the dominance results meant the percentage of ALFF/FCD contributing to timescales where higher number meant higher association with timescales.

The mean timescale maps across healthy subjects in dataset 1 and dataset 2 are drawn in Figure 1. In accordance with a previous study (Watanabe et al., 2019), both dataset 1 and dataset 2 (only HCs) presented similar whole-brain patterns of timescales with longer timescales in frontal and parietal cortices and shorter timescales in other regions such as sensorimotor and visual areas (Figure 1). The spatial correlation between dataset 1 and dataset 2 (only HCs) was *r* = 0.783 (*p* < 0.05, permutation test). As there were two definitions of timescale, we calculated the spatial correlation between mean maps of the two definitions in dataset 1 and dataset 2 (only HCs). The landscapes of these two definitions were in good agreement (dataset 1, *r* = 0.961, *p* < 0.05 for permutation test; dataset 2, *r* = 0.893, *p* < 0.05 for permutation test) (Supplementary Figure 2). We observed significantly negative correlation between age and timescales only in dataset 1 (Supplementary Figure 3), suggesting that the timescales might be related to normal brain aging; results of HCs in dataset 2 were not significant, possibly due to the limited sample size. In addition, timescales in regions such as the left inferior temporal gyrus, sensorimotor cortex, and left middle frontal gyrus presented significantly negative correlation with educational level stating its potential role in the landscape of educational level in HCs of dataset 2 (Supplementary Figure 4).

Then, we assessed the association between intrinsic timescale gradient with common resting-state functional indexes including FCD and ALFF using dominance analysis. Results revealed the relative importance of each predictor (collective *R*^2^ = 0.4528, long-range FCD = 29.19%, global FCD = 27.64%, local FCD = 16.05%, ALFF = 27.11%), suggesting that long-range and global FCD contributed the most to intrinsic neural timescales. These results were validated in HCs of dataset 2 (Supplementary Table 3).

### Altered Timescales in Depression

**FIGURE 2:**
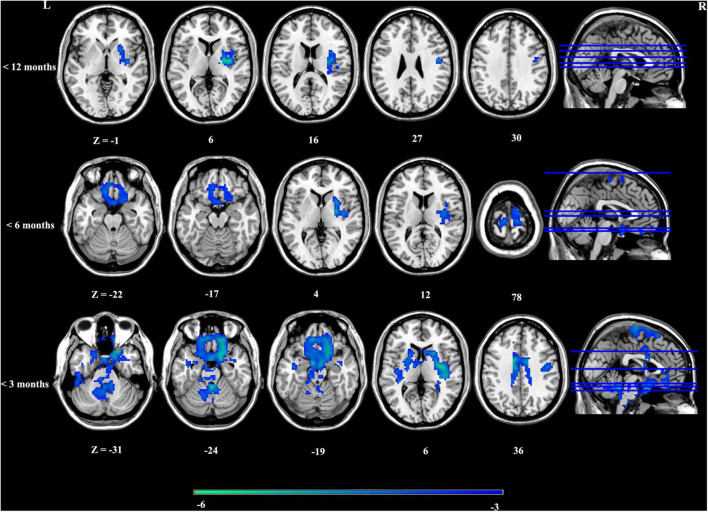
Stage-specific aberrance of timescales in depression.

Overall, there was no significant aberrance of timescales in patients with depression across stages according to illness duration. Whereafter, we investigated altered timescales in patients at different stages according to illness duration. Timescales in patients with depression presented stage-specific aberrance. In particular, patients presented shorter timescales in regions including right anterior insula extending to right putamen only at the first stage (<12 months). To further explore whether this aberrance occurred at disease onset, we investigated timescale aberrance in patients with shorter illness duration (<3/6 months). Regions such as ventral medial prefrontal cortex vmPFC/subgenual PFC, dorsal ACC, dorsal lateral PFC, the bilateral nucleus accumbens (NAcc), the striatum, and the bilateral insula presented decreased timescales in patients with an illness duration of less than 3/6 months. With the prolongation of the disease course, the timescale alterations gradually faded away (Figure 2 and Supplementary Table 4). In addition, to further explore the relationship between two definitions of timescales, the same statistical procedures were done in the second definition of timescales (half maximum of the ACF), and the results are included in Supplementary Figure 5. These results confirmed good consistency of altered intrinsic timescale gradient with different definitions.

To exclude the possibility that the gradual reduction of timescales resulted from the samples used in the current study, we also inquired whether another functional index also presented stage-specific aberrance. For the reason mentioned above, ALFF was chosen. As a result, patients with depression did not present gradual ALFF aberrance in depression (Supplementary Figure 6).

### Relationship to Receptor/Transporter Densities

**FIGURE 3:**
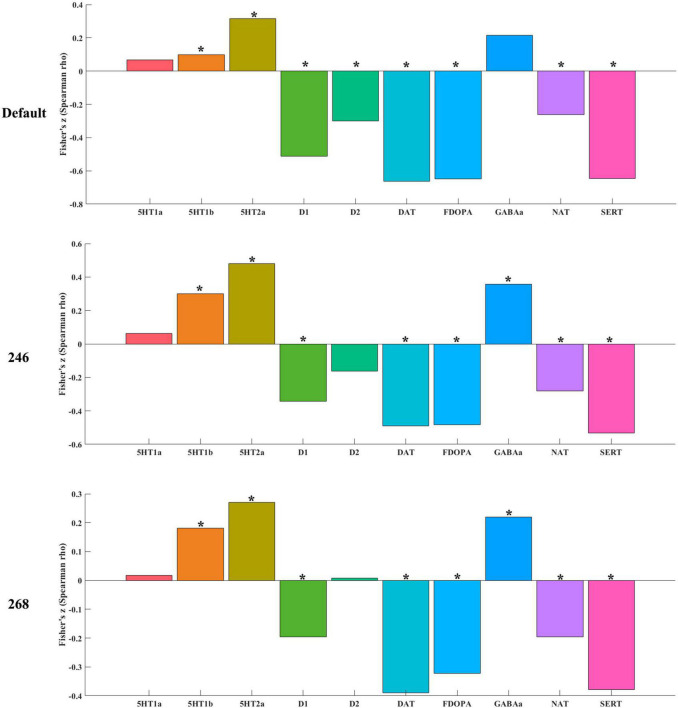
The association between timescale aberrance with receptor/transporter densities. The “*” represented that the correlation was significant (*p* < 0.05 for permutation, FWE corrected).

Note: 5-HT1a, 5-HT subtype 1a; 5-HT1b, 5-HT subtype 1b; 5-HTaa, 5-HT subtype aa; D1, dopamine D1; D2, dopamine D2; DAT, dopamine transporter; F-DOPA, dopamine synthesis capacity; NAT, noradrenaline transporter; SERT, serotonin transporter.

Altered timescales were significantly correlated (*p* < 0.05 for permutation, FWE corrected) with seven receptor/transporter densities (Figure 3 and Supplementary Table 5) including 5-HT2a (5-HT subtype 2a), D1 (dopamine D1), DAT (dopamine transporter), F-DOPA (dopamine synthesis capacity), NAT (noradrenaline transporter), and SERT (serotonin transporter). These results were validated with a different brain atlas (Figure 3). In addition, to explore whether the correlation results were specific to altered timescales, we also calculated spatial correlation between ALFF differences of depression with receptor/transporter densities using default neuromorphometrics atlas. As a result, there was no significant spatial correlation between ALFF aberrance and receptor/transporter densities, hinting that the association was specific to timescale aberrance in depression.

### Cortical Gene Expression Related to Altered Timescales in Depression

As timescales presented stage-specific aberrance and the differences faded away as the progression of illness in depression, we calculated the spatial correlation between gene expression with timescale differences in patients with an illness duration of less than 3 months (see before). As a result, 865/264 genes presented positive/negative correlation with the unthresholded timescale differences in patients with depression whose illness duration was less than 3 months (see section “Supplementary Methods”).

### Enrichment Pathways Associated With Altered Timescales of Depression

**FIGURE 4:**
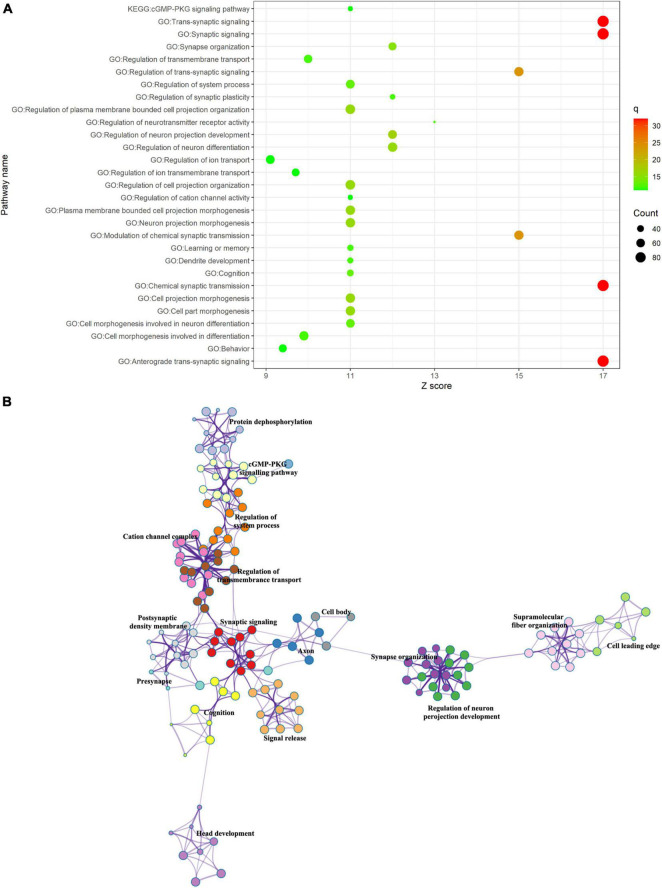
Functional enrichment of gene transcripts. **(A)** Top 30 enrichment terms of positive correlation genes. The size of the circle represented the number of genes enriched in a given term. The color bar represented the significance of a given term. **(B)** Metascape enrichment network visualization. Each term was represented as a circle node where its size is proportional to the number of genes enriched in the term and its color represented cluster identify.

We performed the GO biological processes and KEGG pathways with the associated gene lists obtained in the previous step using Metascape. The top 30 significant GO biological processes, such as “*Trans*-synaptic signaling,” “synapse signaling,” “synapse organization,” “dendrite development,” and “cognition,” and one KEGG pathway, “cGMP-PKG signaling pathway,” were identified (Figure 4) for the positive correlation gene list. Regulation of neurotransmitter receptor activity such as “glutamatergic synapse,” “dopaminergic synapse,” “glutamate receptor signaling pathway,” and “regulation of neurotransmitter secretion” was also identified (see section “Supplementary Methods”). These enrichment terms were clustered into clusters such as synaptic signaling, synapse organization (e.g., dendritic spine morphogenesis and dendrite development), regulation of transmembrane transport, and head development (e.g., hippocampus development and limbic system development) (Figure 4).

Then, we investigated shared enrichment terms between the previously reported polygenic risk for depression and the positive correlation gene list by performing a multi-gene list meta-analysis (Zhou et al., 2019). As a result, we found 11 common pathways. The enrichment pathways included “synaptic signaling,” “synapse organization,” “cell–cell adhesion *via* plasma membrane adhesion molecules,” and “dendrite development” (Supplementary Figure 7).

## Discussion

In this study, we investigated altered intrinsic neural timescale gradient in patients with depression and its possible underlying molecular and transcriptional signatures. Timescales presented stage-specific aberrance in depression. Specifically, patients at the beginning of illness (illness duration <3 months) presented shorter timescales in regions including vmPFC, ACC, the bilateral nucleus accumbens (NAcc), the striatum, and the bilateral insula. As the illness advanced, the difference faded away (disappeared when illness duration ≥12 months). Moreover, the shorter timescales at the beginning of depression were associated with receptor/transporter densities including 5-HT2a, D1/2, DAT, F-DOPA, NAT, and SERT, suggesting the underlying molecular basis of timescale aberrance and providing clues to treatment. Then, we identified timescale aberrance-related genes ontologically enriched for synapse-related and neurotransmitter (receptor) terms elaborating the underlying transcriptional basis of timescale aberrance. These findings revealed atypical intrinsic timescale gradient in depression and bridged the gap between neuroimaging, transcriptome, and neurotransmitter information facilitating an integrative understanding of depression.

### Stage-Specifically Shorter Intrinsic Timescales in Depression

Patients with depression presented stage-specifically shorter timescales according to illness duration in regions including vmPFC, ACC, the bilateral NAc, the striatum, and the bilateral insula. The shorter timescales were only observed in patients with illness duration less than 12 months and then faded away as illness advanced. Converging lines of evidence confirmed that depression was a neuroprogressive illness (Kendler et al., 2001; Moylan et al., 2013); the morphometric alteration of critical brain regions was related to illness progression information (such as illness duration) (Frodl et al., 2003; McKinnon et al., 2009; van Eijndhoven et al., 2009; Alexander-Bloch et al., 2013; Chen V. C. et al., 2016). Consistent with this notion, we observed stage-specifically shorter timescales in patients with illness duration less than 12 months, and even 3 months, suggesting that shorter timescales occurred at the beginning of the disease. In our previous study, we identified that higher brain age was also stage-dependent (Han et al., 2021). This stage-specific aberrance might explain inconsistent findings in depression (Chen Z. et al., 2016). Note that the insignificant timescale aberrance in patients with longer illness duration did not necessarily mean the remission of depression for the reason that we did observe a significant difference in the total score of HAMD (*p* = 0.139, *F* = 2.00) across stages. Regions presenting shorter timescales were found to be related to blunted processing of incentive salience, weak reward source memory, and reinforcement learning underlying the anhedonia in depression (Whitton et al., 2015; Alloy et al., 2016; Han et al., 2020). The shorter timescales of these regions might be associated with inefficient responsiveness to rewards in depression (Whitton et al., 2015; Alloy et al., 2016). On the other hand, dorsal lateral PFC, subgenual PFC, and dorsal ACC belonged to dorsal systems inhibiting amygdala activity in the unstressed state (Phillips et al., 2008). The reduced neuronal size and diminished dendritic arborization in the dorsal system were found in depression (Jaako-Movits et al., 2006; Drevets et al., 2008). The chronic stress could affect the gene expression of monoamine (serotonin)-glutamate/GABA and subsequently affected the E/I balance (Dygalo et al., 2020). Consistent with some ideas, we identified the shorter timescales in the dorsal system resulting from imbalance of the excitation–inhibition (E/I) ratio (Wengler et al., 2020), suggesting the inability of the dorsal system to regulate stress response in depression (Phillips et al., 2008).

There were two possible interpretations for the stage-dependent timescale alteration in depression. First, this phenomenon might mirror the transition from a clinically unstable period, with large variability in functioning, to a relatively stable period, when patients have reached a plateau in functioning (Davidson and McGlashan, 1997; van Haren et al., 2003). Another possible interpretation was that shorter timescales might be associated with primal brain dysfunction in depression. Here, we preferred the latter one. Multimodal lines of evidence convergently indicated that depression was a neuroprogressive illness (Kendler et al., 2001; Moylan et al., 2013). Even patients with depression suffering from only one depressive episode also displayed characteristics of a progressive illness (Moylan et al., 2013). As the disease prolonged, brain tissue damage and physiological functioning gradually changed, which underpinned symptomatology and functional decline over time (Moylan et al., 2013). Our previous results (under review) revealed that progressive morphological alteration might originate from regions like vmPFC and then expand to other regions in depression. Similar progressive morphological alterations such as advanced illness were observed in schizophrenia (Jiang et al., 2018), epilepsy (Zhang et al., 2017), and generalized anxiety disorder (Chen et al., 2020). The original dysfunction might be of great significance to the pathogeny and the treatment of depression. Actually, early treatment of patients with depression is usually accompanied with better outcome of antidepressant treatment and remission, and the reverse was also true. For example, a longer duration of untreated illness was reported to have an unfavorable effect on the subsequent course of the illness (e.g., higher number of recurrences) (Altamura et al., 2007, 2008; Li et al., 2021). A shorter duration of untreated illness was related to better remission of depression and somatic symptoms (Bukh et al., 2013). On the other hand, a longer duration of untreated illness was found to be associated with a greater severity and a lower improvement percentage (Hung et al., 2015; Kraus and Kadriu, 2019). What is more, we found that the stage-specific aberrance might be specific to an intrinsic timescale and did not result from the sample used in the current study. In summary, the shorter timescales at the beginning of disease might reflect initial functional aberrance and mean a lot to subsequent treatment.

### Molecular Architecture of the Shorter Timescales

To explore the potential neurophysiological mechanism underlying the shorter timescales observed in depression helping to facilitate a link to treatment (Chen et al., 2021), we calculated spatial correlation between maps of a variety of neurotransmitter systems with that of timescale aberrance (Dukart et al., 2021). In line with the monoamine hypothesis (Liu et al., 2018), shorter timescales were associated with monoamine neurotransmitters including serotonin, noradrenaline, and dopamine at the same time. It was not unexpected that the timescale differences were associated with serotonin and noradrenaline neurotransmissions because of their fatal roles in pathogenesis (Hamon and Blier, 2013) and the first-line treatment of depression by inhibiting the action of the serotonin/noradrenaline transporter to reduce reuptake of serotonin/noradrenaline (Pirker et al., 1995). Consistent with studies showing that 5-HT2a and SERT were decreased in patients with depression (Kambeitz and Howes, 2015; Steinberg et al., 2019), we observed that the substrate of timescale differences might be related to 5-HT2a and SERT. The reason might be that the 5-HT2A receptors have both excitatory and inhibitory roles underlying the potential biological mechanism of timescale hierarchies (Chaudhuri et al., 2015). The association between SERT binding and rsFC (Beliveau et al., 2015) and dysfunction of SERT binding could result in altered functional connectivity in depression (Han et al., 2019) followed by altered timescales in depression. In addition, we found that the timescale aberrance might be also related to dopaminergic neurotransmission. Dopaminergic neurotransmission playing an essential role by rewarding prediction error (Hollerman and Schultz, 1998; Bayer and Glimcher, 2005) and mediating motivational drive by the attribution of incentive salience to reward-related stimuli (Berridge, 2007) was also related to anhedonia and amotivation in depression (Mayberg et al., 2005). Reduced DAT density in the central and basal nuclei of the amygdala was found in a post-mortem study (Klimek et al., 2002). The association between timescale differences with dopaminergic neurotransmission suggested that shorter timescales of these regions might result in inefficient responsiveness to rewards (Whitton et al., 2015; Alloy et al., 2016). Engaging additional targets (e.g., DA) could help patients with residual symptoms and treatment-resistant depression (Blier, 2016). Combining with these findings, our results revealed the role of dopaminergic neurotransmission in timescale aberrance. What is more, the validation results confirmed the robustness (selection of different atlas) and specificity (Compared with ALFF) of association between timescale aberrance and neurotransmitter information. These results suggested a potential neurophysiological mechanism underlying the shorter timescales observed in depression, providing clues to treatment.

### Altered Timescale-Related Gene Expressions Enriched for Functional Annotations

We identified genes whose expression pattern presented significantly spatial (positive/negative) correlation with timescale aberrance and their ontology terms elaborated the underlying transcriptional basis of timescale aberrance. Consistent with the monoamine hypothesis in depression (Liu et al., 2018), positive correlation genes related to timescale difference were significantly enriched in monoamine neurotransmitter-related GO biological processes/KEGG pathways including neurotransmitter secretion, transport, and receptor activity/complex. These results corresponded with the aforesaid findings about monoamine aberrance, suggesting transcriptional mechanisms of association between timescale difference and monoamine neurotransmitters. In recent years, the synaptic dysfunction hypothesis that depression was caused by disruption of homeostatic mechanisms controlling synaptic plasticity (Duman and Aghajanian, 2012) has been proposed in consideration of the moderate and delayed effectiveness of the widely prescribed serotonin selective reuptake inhibitors (SSRIs) (Trivedi et al., 2006) and rapid antidepressant actions of ketamine in treatment-resistant depressed patients (Berman et al., 2000; Zarate et al., 2006). Deficits of excitatory glutamate neurons and inhibitory GABA interneurons resulted in the vulnerability of these major neurotransmitter systems followed by dendritic atrophy and spine loss in neurons of the hippocampus and prefrontal cortex (Qiao et al., 2016; Duman et al., 2019). Dendrite complexity and synaptic density can also be increased after treatment with antidepressants (Li et al., 2010; Li et al., 2011; MacQueen and Frodl, 2011). In our study, the timescale difference-related genes were enriched in terms of the charge of the balance of excitation and inhibition including glutamatergic synapse, transmission, receptor signaling pathway, GABAergic synapse, regulation of NMDA receptor activity, and G protein-coupled receptor signaling pathway. In fact, the spatial correlation (*p* = 0.024 uncorrected) between timescale aberrance and GABAa (gamma-aminobutyric acid) was also observed in the current study. These results elaborated possible transcriptional basis underlying the altered intrinsic timescale gradient in depression and provided new lines of evidence supporting the synaptic dysfunction hypothesis. In addition to the overlapping ontology terms with that in GWAS in depression, the multi-gene list results stated that timescale difference-related genes were reliable and sensitive, providing additional function-related enrichment information for depression.

There were several limitations to be considered. First, the timescale differences were obtained on a single dataset. However, the stage-specific aberrance was also observed in accelerated brain aging GMV in our previous study and might not result from sample selection (Han et al., 2021). Second, there was a substantial variation across subjects reflecting the individual susceptibility of specific receptor systems (Dukart et al., 2021); future studies should use simultaneous PET and MRI to provide more direct evidence. Third, patients enrolled in our study were under a depressive state. Whether the timescale difference was differently altered in various mood states, such as remitted state (Rive et al., 2015), could be tested in further studies. Fourth, only cross-sectional data were included in this study, and future studies could explore whether altered intrinsic timescale gradient returned to normal with antidepressant treatment especially for fast-acting antidepressants (Li, 2020).

## Conclusion

This study revealed atypical intrinsic timescale gradient for the first time. In virtue of brain-wide gene expression and molecular imaging atlases, we investigated possible underlying molecular and transcriptional basis of timescale aberrance linking transcriptome, neurotransmitter information, and neuroimaging findings in depression. These results consistently supported the synaptic dysfunction hypothesis and promoted an integrative understanding of hierarchical dynamics aberrance in depression.

## Data Availability Statement

The raw data supporting the conclusions of this article will be made available by the authors, without undue reservation.

## Ethics Statement

The studies involving human participants were reviewed and approved by the Research Ethical Committee of The First Affiliated Hospital of Zhengzhou University. The patients/participants provided their written informed consent to participate in this study.

## Author Contributions

SH and JC designed the study. RZ, SL, BZ, and YJ collected the data. SH and YC analyzed the data. SH and YZ drafted the work. YW, CW, JP, and HL revised the draft. All authors contributed to the article and approved the submitted version.

## Conflict of Interest

The authors declare that the research was conducted in the absence of any commercial or financial relationships that could be construed as a potential conflict of interest.

## Publisher’s Note

All claims expressed in this article are solely those of the authors and do not necessarily represent those of their affiliated organizations, or those of the publisher, the editors and the reviewers. Any product that may be evaluated in this article, or claim that may be made by its manufacturer, is not guaranteed or endorsed by the publisher.

## Footnotes

1
http://rfmri.org/dpabi

2
https://github.com/dominance-analysis/dominance-analysis

3
https://github.com/juryxy/JuSpace

4
http://human.brain-map.org/

5
https://istbi.fudan.edu.cn/lnen/info/1173/1788.htm
