# Supplementary material for: Integrative Functional, Molecular, and Transcriptomic Analyses of Altered Intrinsic Timescale Gradient in Depression
Source: Front Neurosci. 2022 Feb 17;16:826609. doi: 10.3389/fnins.2022.826609 (PMC8891525; doi:10.3389/fnins.2022.826609)
Supplement: Supplementary file 1 [file Data_Sheet_1.pdf]

Supporting information for
Integrative functional, molecular and transcriptomic analyses of altered intrinsic timescale gradient in depression

Supplementary Method

Dataset 1

Dataset 1 come from Southwest University Adult Lifespan Dataset (SALD) study available for research purposes through the International Data-sharing Initiative (http://fcon_1000.projects.nitrc.org/indi/retro/sald.html). This dataset included 494 healthy participants (308 female, 187 male, age range 19-80). The exclusion criteria included MRI-related exclusion criteria, current psychiatric/neurological disorders, use of psychiatric drugs in the past three months prior to scanning and so on. More detailed description about the subject information and data acquisition parameters, please see [1]. Subjects were excluded if the translational and rotational displacement exceeded 3.0 mm or 3.0° (see date preprocessing), 25 subjects were excluded according to this principle.

Dataset 2

Dataset 2 included 121 HCs and 191 patients with depression. patients with depression were recruited from out-patient services of Department of Psychiatry, the First Affiliated Hospital of Zhengzhou University since January 2015. Patients were diagnosed according to Diagnostic and Statistical Manual of Mental Disorders, Fourth Edition (DSM-IV) for depression. This procedure was done by one chief physician and one well-trained psychiatrist. All patients included in the current study were never treated first-episode. The exclusion criteria included: (1) Comorbidity of other mental/psychotic disorders; (2) Previous episodes of manic symptoms. The clinical states of the patients were evaluated using the 24/17-items Hamilton Depression scale (HAMD).

Healthy controls (HCs) were recruited from the community through poster advertisement. None of them presented a history of serious medical or neuropsychiatric illness or a family history of major psychiatric or neurological illness in their first-degree relatives.

Patients and HCs were all Han Chinese and right handedness. In addition, participants (both HCs and patients) included would be excluded if they met one of the following exclusion criteria: (1) Taking drugs such as anesthesia, sleeping and analgesia in the past 1 month. (2) Substance abuse; (3) A history of brain tumor, trauma, surgery, or other organic body disease; (4) Suffering from cardiovascular diseases, diabetes, hypertension; (5) Contraindications for MRI scanning including fixed dentures, metal braces, artificial heart valves and other metal foreign bodies in the body. (6) Other structural brain abnormalities revealed by MRI scan.

Written informed consents were obtained from all participants before scanning. The study was approved by the research ethical committee of the First Affiliated Hospital of Zhengzhou University.

Scan acquisition

Dataset 1

During the resting-state MRI scan, the subjects were instructed to lie down, close their eyes, and rest without thinking about any specific thing but to refrain from falling asleep. The 8- min scan of 242 contiguous whole-brain resting-state functional images was obtained using gradient echo echo-planar-imaging (GRE-EPI) sequences with the following parameters: slices = 32, repetition time (TR)/echo time (TE) = 2000/30 ms, flip angle = 90, field of view (FOV) = 220 × 220 mm, thickness/slice gap = 3/1 mm, and voxel size = 3.4 × 3.4 × 4 mm^3 and total

Dataset 2

The MRI data were acquired using a 3-T GE Discovery MR750 scanner. An eight-channel prototype quadrature birdcage head coil fitted with foam padding was used to minimize head movement. Participants were asked to remain motionless, keep their eyes closed, not think of anything and let the mind wander. Functional images were obtained using an echo-planar imaging sequence. The parameters were as follow: TR/TE = 2000/40 ms, 32 slices, matrix size = 64 × 64, voxel size = 3.75 × 3.75 × 3.2 mm3, flip angle = 90°, slice thickness = 4.0 mm, gap = 0.5 mm and total 180 volumes.

Imaging data processing

Functional images were preprocessed using Data Processing & Analysis for Brain Imaging (DPABI, http://rfmri.org/dpabi)[2]. The following steps were done: Remove of the first 10/5 (dataset 1/dataset 2) volumes, slice timing and realignment. Then images were normalized to the standard EPI template (resampled into 3 × 3 ×3 mm3). Signal-to-noise ratios (SNR0) was determined by obtaining the mean signal and SD for a given slice across the BOLD run to control image quality. Subjects were excluded if the translational and rotational displacement exceeded 3.0 mm or 3.0°. Twenty-three subjects of dataset 1 were excluded according to this standard. Then images were smoothed using 6 × 6 × 6 mm^3 full width at half maximum (FWHM) Gaussian kernel and detrended to reduce low-frequency drift. Band-pass filtered (0.01 - 0.1 Hz) to remove high-frequency physiological noise. Friston 24 motion parameters [3], white matter signal and cerebrospinal fluid signal were regressed out as nuisance covariates. Specially, the global signal was not regressed in the current study for the reason that the variance of global signal along with its topography were found altered mental disease including depression [4-6]. The variance of global signal was calculated and compared between patients and HCs (Figure S1). Despiking, we replaced the outliners with the best estimate using a third-order spline fit to clean the portions of time course. Outliners were detected based on the median absolute deviation, as implemented in 3dDespike (http://afni.nimh.nih.gov/afni) [7]. The mean frame-wise displacement (FD) was also calculated [8, 9].

Identifying factors affecting timescales

As a newly proposed index, factors affecting timescales remained unclear. To explore these factors, we investigated whether factors such as gender (female vs. male), age, motion movement and education level could affect intrinsic timescale gradient. Specially, to explore effect of gender on the landscape of timescale, we compared timescale map of female with that of male using two sample t test. As for motion movement, education level and age, we adopted multiple linear regression equipped in SPM12 between factor (motion movement, education level or age) and timescale maps respectively. Results were corrected for multiple comparison (voxel-wise $p < 0.001$,

cluster-level $p < 0.05$; Gaussian random field (GRF) corrected). This procedure was done in discovery cohort (dataset 1) and validated in replication cohort (HCs of dataset 2), results reported were based on the discovery cohort.

Dominance analysis

As a method for evaluating the relative importance of predictors, dominance analysis estimated the relative importance of predictors by constructing all possible combinations of predictors and quantifying the relative contribution of each predictor as additional variance explained (i.e. gain in R^2) by adding that predictor to the models (Azen and Budescu, 2003; Budescu and David, 1993). Specifically, for n predictors we have $2^n - 1$ models that include all possible combinations of predictors. The incremental R^2 contribution of each predictor to a given subset model of all the other predictors was then calculated as the increase in R^2 due to the addition of that predictor to the regression model. Here we constructed dominance model with local, long-range and global FCD and ALFF maps as independent variables and timescales map as the dependent variable to quantify the distinct contribution of each factor using dominance analysis.

Gene expression profile

Two of the brains were with both hemispheres and four with the left hemisphere. The number of anatomic samples obtained from each brain varied from 363 to 946. We followed the pipeline provided by the Allen Brain Atlas to obtain and process the raw expression data (see http://www.brain-map.org/api/examples/examples/doc/wgcna/preprocessData.R.html). To get the expression value of genes from their corresponding probes, the 'collapseRows' function from the WGCNA package was employed to pick the probe with the highest average expression to represent that gene. In total, 3695 unique anatomic samples with 20,738 gene expression profiles were obtained (details of AHBA's microarray information/data normalization: http://help.brain-map.org/display/humanbrain/documentation/). To further remove individual differences and pool all the AHBA samples from different subjects together to provide voxel-level genetic knowledge, a normalization procedure was applied: for each given gene in any individual, expressions were normalized by extracting the median of the gene's expression across all samples of the individual and were divided by the median. Then, for each AHBA tissue sample, we created a 6 mm sphere region of interest (ROI) in the MNI volume space centered on its MNI centroid coordinate. Finally, 3695 ROIs with their corresponding normalized gene expression profiles were used in our following analysis.

Supplementary Results

Factors affecting landscape of the timescales

Next, we explored factors affecting landscape of timescales. We did not observe significant difference between female and male subjects in dataset 1 and HCs of dataset 2 suggesting gender did not affect landscape of timescales. There was no significant correlation between mean FD and timescales in in dataset 1 and HCs of dataset 2. We observed significantly negative correlation between age and timescales only in dataset 1 (Figure S2) suggesting the timescales might be related to normal brain aging, results of HCs in dataset 2 were not significant the reason could be the limited sample size. In addition, timescales in regions such as left inferior temporal gyrus, left middle frontal

gyrus and sensorimotor cortex presented significantly negative correlation with educational level stating its potential role in landscape of educational level in HCs of dataset 2 (Figure S4). The results were corrected for multiple comparison (voxel-wise $p < 0.001$, cluster-level $p < 0.05$; Gaussian random field (GRF) correction).

Supplementary Discussion

The landscape and affecting factors of intrinsic neural timescales

Hierarchical dynamics (or intrinsic timescale gradient) represented a general, intrinsic organizing principle of mammalian brains supported by multimodal evidences (Raut and Snyder, 2020). The hierarchy was deeply embedded within the functional architecture of neocortex (Burt et al., 2018; Chaudhuri et al., 2015; Demirtaş et al., 2019) and topographically mirrored in striatum, thalamus, and cerebellum (Raut and Snyder, 2020). Consistent with findings of these studies, we found that intrinsic timescales were repeatable organized along spatial gradients that longer timescales in frontal and parietal cortices and shorter timescales in other regions such as sensorimotor and visual areas across two independent datasets. The biological basis of intrinsic timescale gradient was hypothesized to emerge through a combination of hierarchically organized fine-scale features (Burt et al., 2018; Demirtaş et al., 2019; Fulcher et al., 2019; Huntenburg et al., 2018; Wang, 2020) including pyramidal cell dendritic spine density(Elston, 2003), the associated degree of recurrent excitation (Chaudhuri et al., 2015) and long-range connectivity (Baria et al., 2013; Chaudhuri et al., 2015; Gollo et al., 2015; Huntenburg et al., 2018). In line with these notions, Takamitsu et al. found that the longer intrinsic timescales were companied with larger gray matter volumes (GMVs) indicating a high density of neurons (Kanai and Rees, 2011), more synapses (Cullen et al., 2010) greater synaptic weights (Perin et al., 2011) in local brain regions. Here, we replicated this result in two datasets (Figure S8) suggesting brain regions with larger GMVs were companied with larger autocorrelations(Perin et al., 2013)(Ikegaya et al., 2004). Rishidev et al. suggested that interregional variation in the level of recurrent excitation could give rise to timescale grandient by large-scale dynamic model (Chaudhuri et al., 2015). In the current study, we found the landsacape of the intrinsic neural timescale was close to that of long-range FCD supporting that the intrinsic timescale gradient was close to long-range connectivity (Baria et al., 2013; Chaudhuri et al., 2015; Gollo et al., 2015; Huntenburg et al., 2018). The dominance results gave more intuitional and comprehensible explain of intrinsic timescale gradient by exploring the association with existing functional indexes for readers. What is more, the present study explored factors affected the landscape of timescales providing information about what factors to be controlled for the following studies.

Table S1. Demographic and Clinical Characteristics of Participants in Dataset 1 and Dataset 2.

	Dataset 1	Dataset 2	
	HCs (N = 469)	HCs (N = 121)	Depression (N = 191)
Male, No. (%)	177 (37.74)	65 (53.72)	95 (49.74)
Age, mean (SD) [range], y	44.89 (17.35) [19-80]	20.89 (5.27) [12-36]	18.10 (4.50) [11-37]
Educational level, mean (SD), y	-	13.50 (4.51)	10.08 (2.11)
Duration of illness, mean (SD), m	-	-	16.10 (19.27)
HAMD score, mean (SD), [range]	-	-	22.38 (5.72) [12-48][a]<br>39.29 (11.68) [20-61][b]
Handedness, right/left	-	121/0	191/0
Age of first onset, y	-	-	16.80 (4.54)
SNR0, mean (SD)	132.14 (19.49)	207.72 (36.82)	193.77 (34.46)
Mean FD, mean (SD)	0.11 (0.06)	0.11 (0.06)	0.11 (0.05)

Abbreviations: HAMD, Hamilton rating scale for depression; [a],17-items HAMD for 167 patients; [b], 24-items HAMD for 28 patients

Table S2. The clinical demographics of patients at different stages and matched HCs.

	Depression				HCs	p
	Stage 1	Stage 2	Stage 3	All		
Age (years), mean ± SD	17.10 ± 3.60	18.17 ± 4.82	18.62 ± 3.65	18.10 ± 4.50	18.72 ± 5.17	0.10[a]
Gender, male : female	46 : 44	24 : 29	24 : 24	96 : 95	44 : 51	0.98[b]
Duration of illness (months), mean ± SD	4.05 ± 2.46	12.89 ± 2.02	39.07 ± 17.73	16.10 ± 19.27	-	-
Years of education, mean ± SD	10.04 ± 2.20	9.77 ± 2.05	9.82 ± 2.06	10.08 ± 2.11	10.42 ± 4.25	0.38[a]
Handedness, right/left	90/0	53/0	48/0	191/0	95/0	-
Age of first onset (years)	16.80 ± 3.60	16.96 ± 4.71	15.47 ± 3.82	16.27 ± 5.34	-	-
SNR0, mean (SD)	196.55 (35.61)	193.60 (32.35)	197.35 (33.18)	193.77 (34.46)	206.71 (36.17)	< 0.01[a]
Mean FD, mean (SD)	0.11 (0.05)	0.12 (0.06)		0.11 (0.05)	0.11 (0.06)	0.98[a]

Note: [a] two-tailed two sample *t* test; [b] Chi-square *t*-test; HC, healthy control; Mean FD, mean frame-wise displacement

Table S3. The dominance results in dataset 2 (only HCs).

collective R^2	long-range FCD	global FCD	local FCD	ALFF
0.618	34.17%	31.35%	19.44%	15.04%

Table S4. Altered timescales in patients with depression.

Stages	Clusters	Voxels	Regions	MNI (x, y, z)	T
Stage 1	1	459	right anterior insula	33, -9, 9	-5.65
(Duration < 12 m)			Right putamen		
Duration < 6 m	1	821	Right putamen	27, -6, 6	-5.36
			Right insula		
			Ventral medial prefrontal cortex		
	2	338	Bilateral precentral gyrus	15, -24, 78	-4.32
			Superior frontal gyrus		
Duration < 3 m	1	6231	Middle cingulate gyrus	-12, 3, 36	-5.46
			Anterior cingulate cortex		
			Superior frontal gyrus		
			Paracentral Lobule		
			Ventral medial prefrontal cortex		
			Cerebelum		
			Bilateral insula		
			bilateral nucleus accumbens		
			dorsal lateral PFC		
			Bilateral putamen		
			Bilateral Thalamus		
	2	826	Medial Frontal Gyrus	6, -12, 78	-4.99
			supplementary motor area		
			Superior frontal gyrus		
			Precentral Gyrus		
			Postcentral Gyrus		

Figure S4. Altered ALFF in patients with all patients with depression. ALFF did not presented stage-specific alteration in patients with depression.

Clusters	Voxels	Regions	MNI (x, y, z)	T
1	361	Left Cerebelum	-12,-48,-42	4.80
2	64	Right Parahippocampa Gyrus	30,-12,-30	4.40
		Right Hippocampus		
3	24	Right amygdala	21,-9,-9	4.38
4	117	Right Precuneus	3,-63,66	-5.34
		Parietal Lobe		

Table S5. Spatial correlation between IDSCNs with receptor/transporter densities for subgroup 1.

Atlas	PET Map	Fisher'z (Spearman rho)	p-value (uncorrected)
Default	5H1a	0.163	0.081
	5H1b	0.174	0.063
	5H2a	0.344	< 0.001
	D1	-0.092	0.321
	D2	-0.138	0.138
	DAT	-0.341	< 0.001
	FDOPA	-0.458	< 0.001
	GABAa	0.232	0.013
	NAT	-0.123	0.188
	SERT	-0.344	<0.001
246	5H1a	0.062	0.336
	5H1b	0.299	<0.001
	5H2a	0.480	<0.001
	D1	-0.342	<0.001
	D2	-0.164	0.011
	DAT	-0.490	<0.001
	FDOPA	-0.482	<0.001
	GABAa	0.358	<0.001
	NAT	-0.282	<0.001
	SERT	-0.532	<0.001
268	5H1a	0.017	0.782
	5H1b	0.182	0.003
	5H2a	0.270	<0.001
	D1	-0.195	0.002
	D2	0.007	0.906
	DAT	-0.389	<0.001
	FDOPA	-0.323	<0.001
	GABAa	0.220	<0.001
	NAT	-0.196	0.001
	SERT	-0.379	<0.001

Figure S1. Decreased variance of global signal in depression.

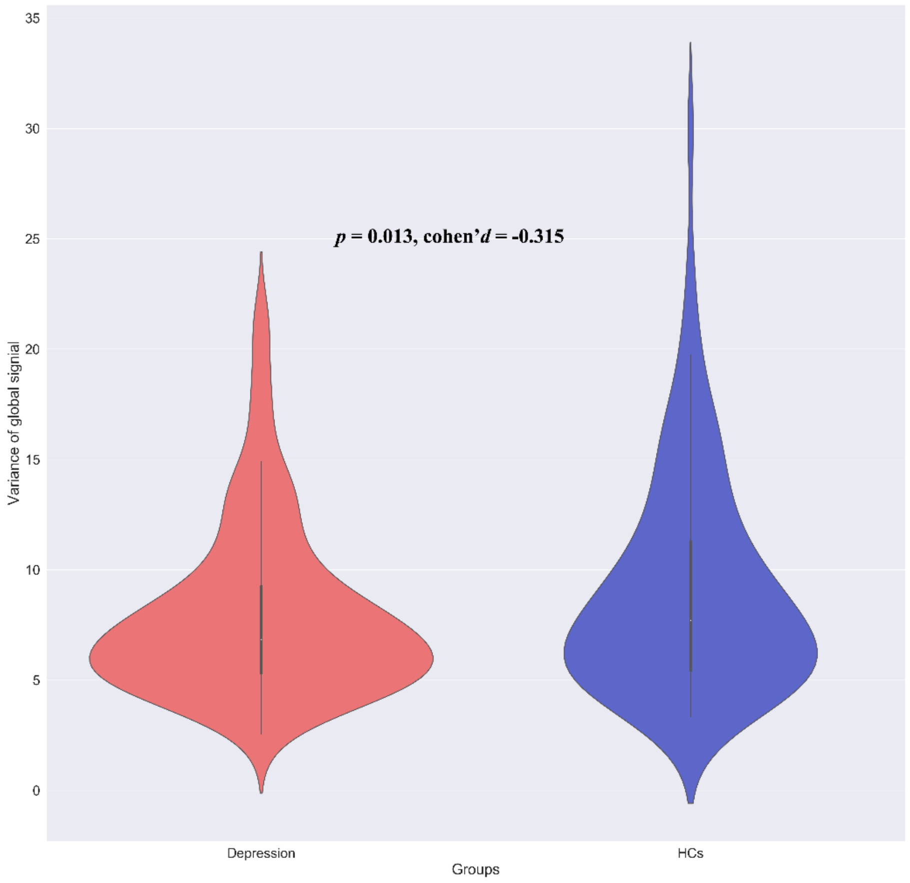

Figure S2. The spatial correlation between two definitions of timescales. The $p < 0.05$ for reported r values (permutation test).

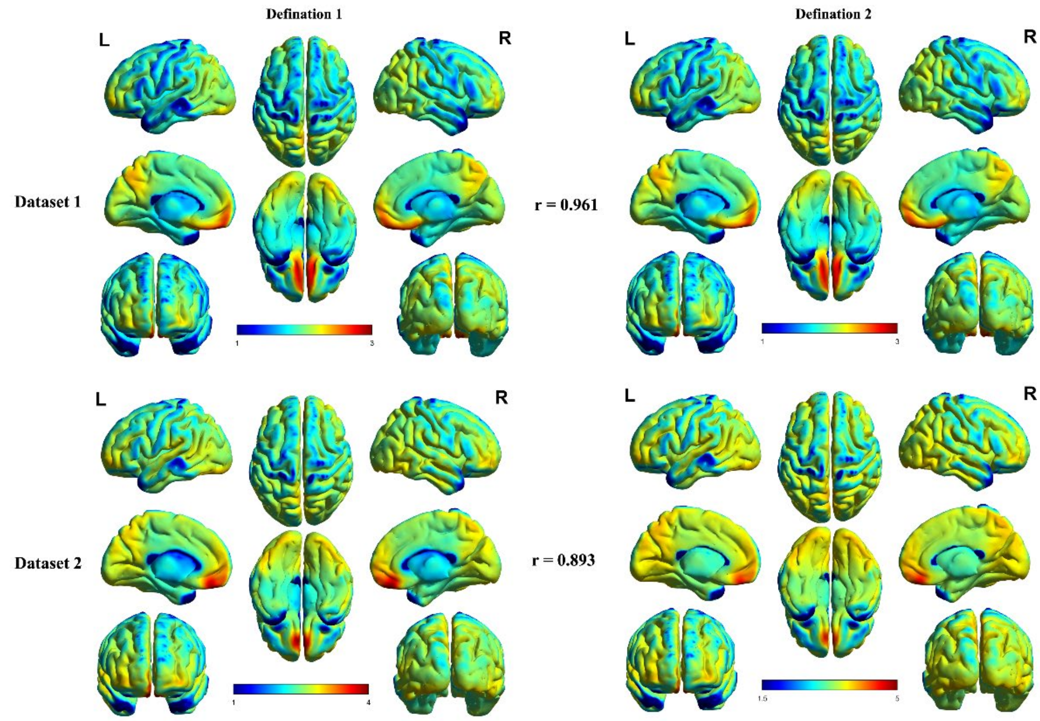

Figure S3. The association between timescales and age. Results were corrected for multiple comparison (voxel-wise $p < 0.001$, cluster-level $p < 0.05$; Gaussian random field (GRF) correction).

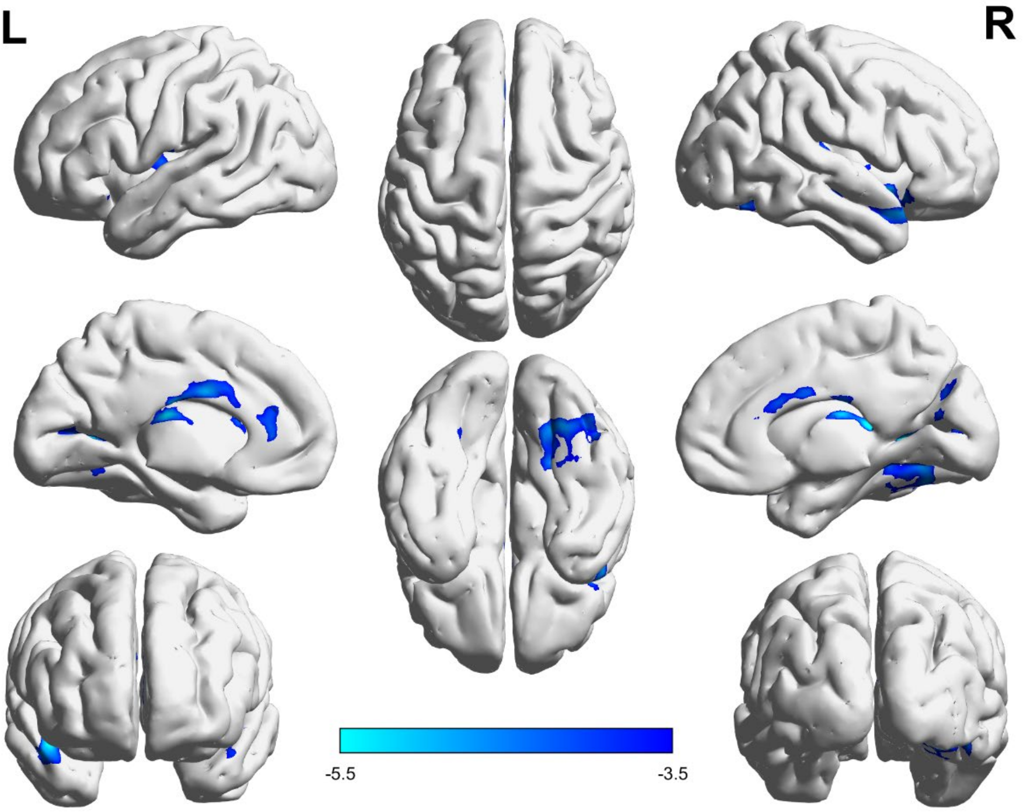

Figure S4. The association between timescales and educational level. Results were corrected for multiple comparison (voxel-wise $p < 0.001$, cluster-level $p < 0.05$; Gaussian random field (GRF) correction).

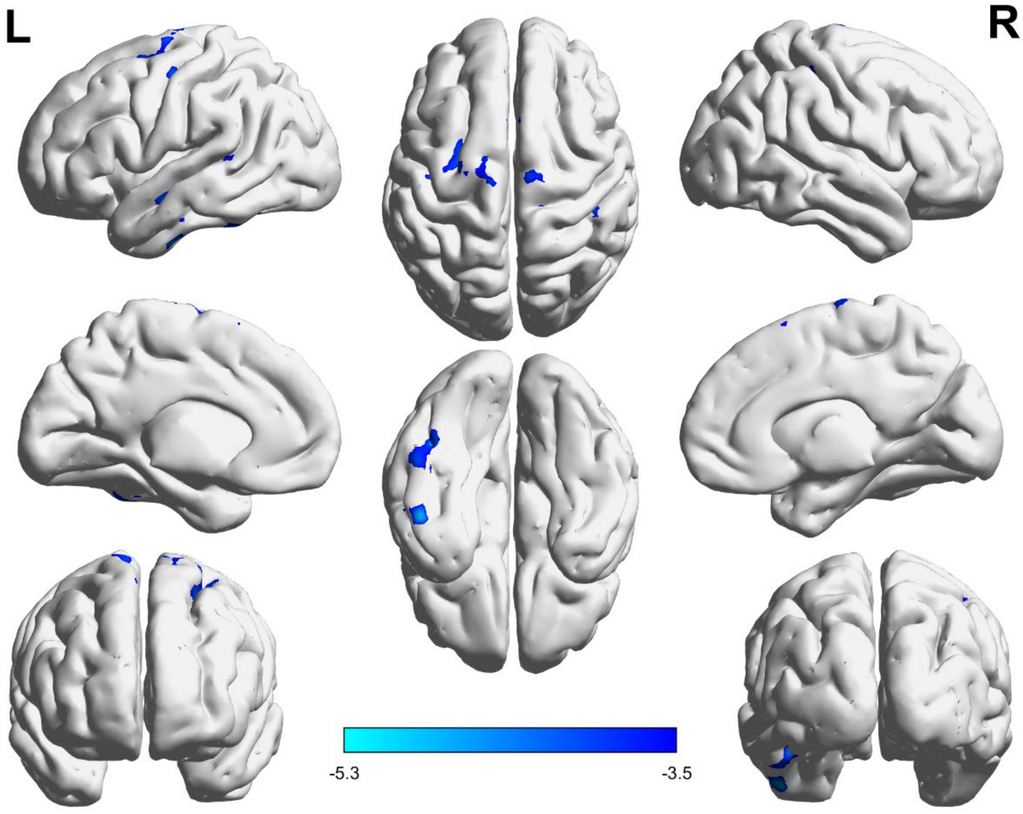

Figure S5. Altered timescales with the second definition in different stages of depression. The number meant its spatial correlation coefficient with that gotten with the first definition. These results confirmed good consistency of altered intrinsic timescale gradient with different definitions.

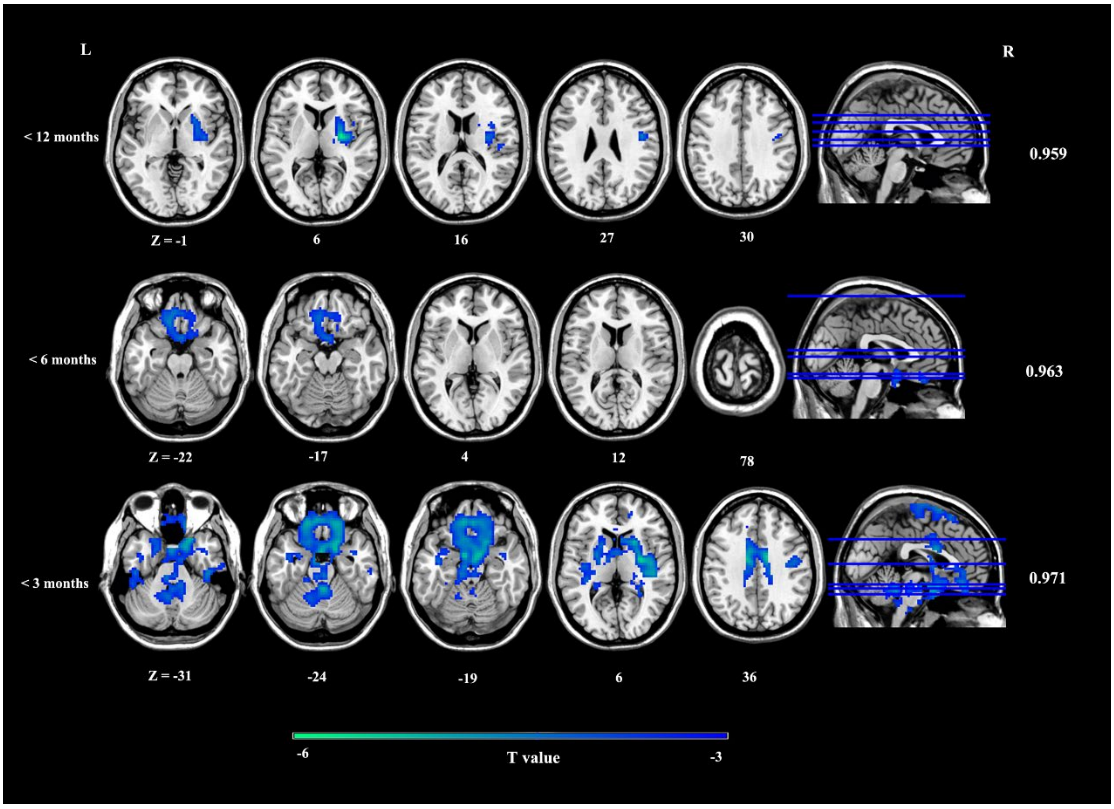

Figure S6. Altered ALFF in depression. We only observed altered ALFF across all stages of depression. Results were corrected for multiple comparison (voxel-wise p < 0.001, cluster-level p < 0.05; Gaussian random field (GRF) correction).

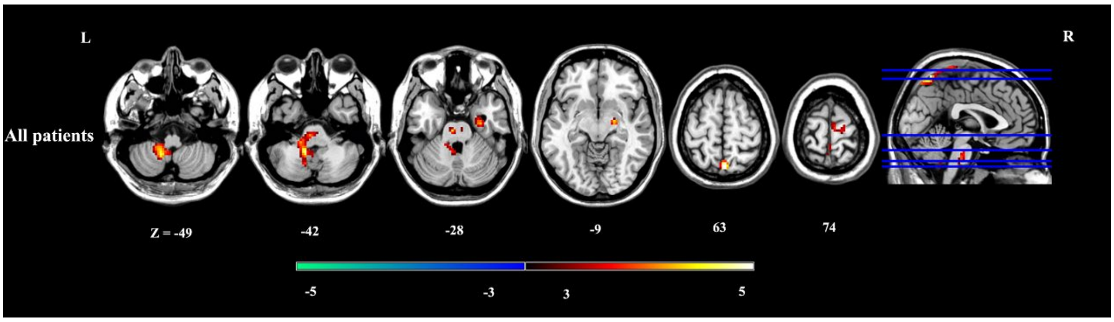

Figure S7. Overlapped ontology terms between discovery cohort and GWAS studies. (A) Enrichment heatmap visualization. The heatmap cells were colored by their p-values where white cells indicated the lack of enrichment for that term in the corresponding genes. (B) Ontology terms. (C) Overlapped distribution of the same enrichment network. The nodes were displayed as pies whose pie sector was proportional to the number of hits originated from a gene list.

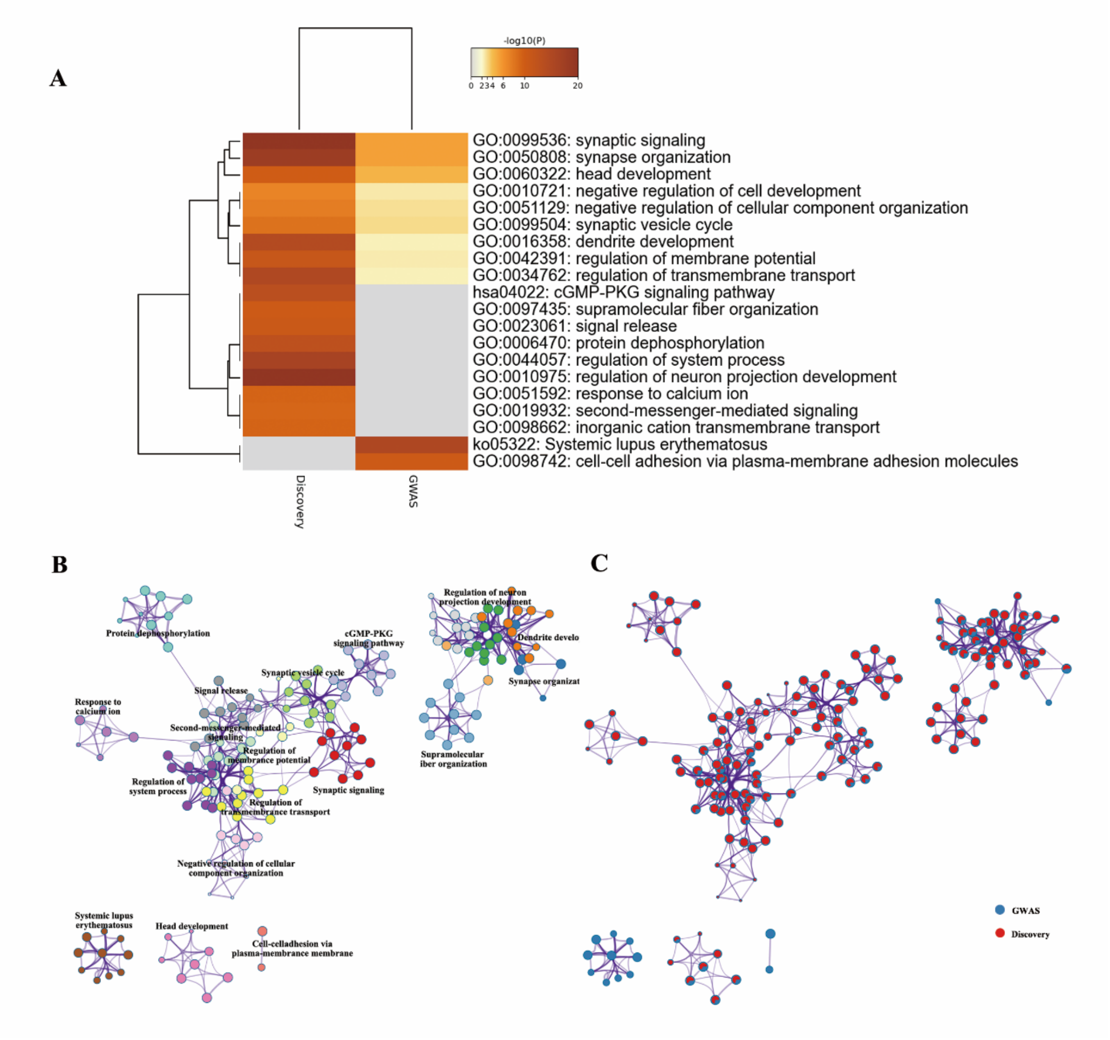

Figure S8. Spatial correlation between timescale and gray matter volume.

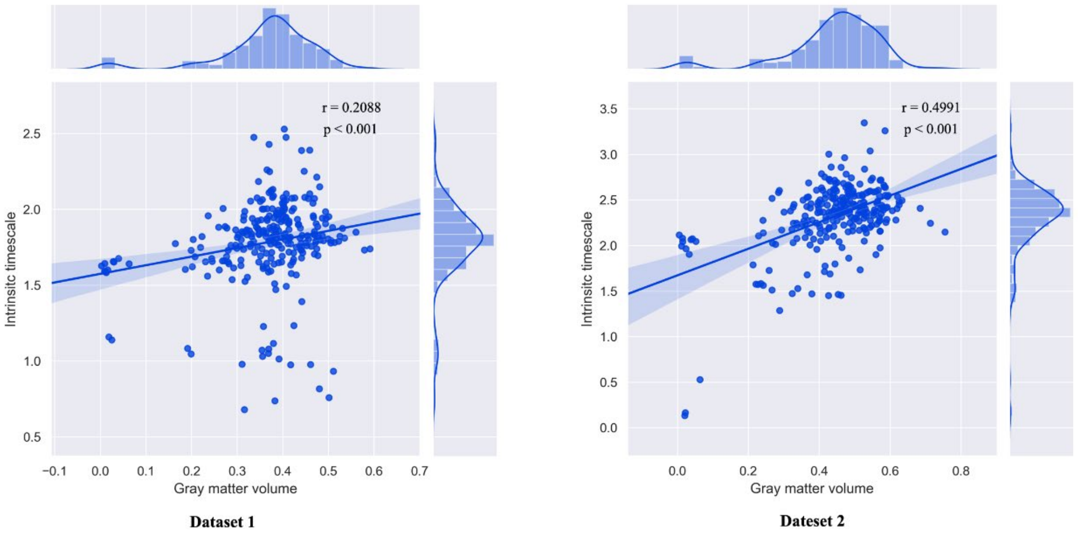

References:

1. Wei, D., et al., *Structural and functional brain scans from the cross-sectional Southwest University adult lifespan dataset.* Sci Data, 2018. **5**: p. 180134.
2. Yan, C.-G., et al., *DPABI: Data Processing & Analysis for (Resting-State) Brain Imaging.* Neuroinformatics, 2016. **14**(3): p. 339-351.
3. Satterthwaite, T.D., et al., *Impact of in-scanner head motion on multiple measures of functional connectivity: Relevance for studies of neurodevelopment in youth.* Neuroimage, 2012. **60**(1): p. 623-632.
4. Han, S., et al., *Decreased static and increased dynamic global signal topography in major depressive disorder.* Prog Neuropsychopharmacol Biol Psychiatry, 2019. **94**: p. 109665.
5. Zhang, J., et al., *Altered Global Signal Topography and Its Different Regional Localization in Motor Cortex and Hippocampus in Mania and Depression.* Schizophr Bull, 2019. **45**(4): p. 902-910.
6. Yang, G.J., et al., *Altered global brain signal in schizophrenia.* Proc Natl Acad Sci U S A, 2014. **111**(20): p. 7438-43.
7. Allen, E.A., et al., *Tracking Whole-Brain Connectivity Dynamics in the Resting State.* Cerebral Cortex, 2014. **24**(3): p. 663.
8. Han, S., et al., *Dysfunctional connectivity between raphe nucleus and subcortical regions presented opposite differences in bipolar disorder and major depressive disorder.* Progress in neuro-psychopharmacology & biological psychiatry, 2018.
9. Han, S., et al., *Resting state functional network switching rate is differently altered in bipolar disorder and major depressive disorder.* 2020. **41**(12): p. 3295-3304.
